# Assessment of social psychological determinants of satisfaction with childbirth in a cross-national perspective

**DOI:** 10.1186/1471-2393-7-26

**Published:** 2007-10-26

**Authors:** Wendy Christiaens, Piet Bracke

**Affiliations:** 1 Department of Sociology, Ghent University, Ghent, Belgium

## Abstract

**Background:**

The fulfilment of expectations, labour pain, personal control and self-efficacy determine the postpartum evaluation of birth. However, researchers have seldom considered the multiple determinants in one analysis. To explore to what extent the results can be generalised between countries, we analyse data of Belgian and Dutch women. Although Belgium and the Netherlands share the same language, geography and political system and have a common history, their health care systems diverge. The Belgian maternity care system corresponds to the ideal type of the medical model, whereas the Dutch system approaches the midwifery model. In this paper we examine multiple determinants, the fulfilment of expectations, labour pain, personal control and self-efficacy, for their association with satisfaction with childbirth in a cross-national perspective.

**Methods:**

Two questionnaires were filled out by 605 women, one at 30 weeks of pregnancy and one within the first 2 weeks after childbirth either at home or in a hospital. Of these, 560 questionnaires were usable for analysis. Women were invited to participate in the study by independent midwives and obstetricians during antenatal visits in 2004–2005. Satisfaction with childbirth was measured by the Mackey Satisfaction with Childbirth Rating Scale, which takes into account the multidimensional nature of the concept. Labour pain was rated retrospectively using Visual Analogue Scales. Personal control was assessed with the Wijma Delivery Expectancy/Experience Questionnaire and Pearlin and Schooler's mastery scale. A hierarchical linear analysis was performed.

**Results:**

Satisfaction with childbirth benefited most consistently from the fulfilment of expectations. In addition, the experience of personal control buffered the lowering impact of labour pain. Women with high self-efficacy showed more satisfaction with self-, midwife- and physician-related aspects of the birth experience.

**Conclusion:**

Our findings focus the attention toward personal control, self-efficacy and expectations about childbirth. This study confirms the multidimensionality of childbirth satisfaction and demonstrates that different factors predict the various dimensions of satisfaction. The model applies to both Belgian and Dutch women. Cross-national comparative research should further assess the dependence of the determinants of childbirth satisfaction on the organisation of maternity care.

## Background

In previous research some determinants of childbirth satisfaction have been proposed, but only a few authors [1-3] considered the multiple determinants within a single study. Social psychological determinants most authors agree about – expectations about childbirth, labour pain, personal control and self-efficacy – are assessed in relation to satisfaction with childbirth in one analysis. In addition, most of the research focuses on single countries. The question of course is, to what extent the results can be generalised between countries, despite huge variation in childbirth delivery practices.

Although Belgium and the Netherlands share a common history, geography and language, health care in general and maternity care in particular are differently organised. The birth systems can be placed in an international context wherein Belgium represents the mainstream obstetric practice characterised by a highly medicalised approach. The Dutch childbirth system, however, is well known for the high rate of home births. Approximately 30% of Dutch pregnant women have a home birth [4] versus less than 2% of Belgian women [5]. In both countries women can attend primary or secondary caregivers. In the primary care system women deliver at home with a midwife, sometimes accompanied by a general practitioner. In secondary care, childbirth takes place in a hospital under supervision of an obstetrician. In the Netherlands, however, primary caregivers function as gatekeepers [6]. They refer women to secondary care in cases of a reduced chance of a normal birth. In Belgium the great majority (more than 98%) of women consult an obstetrician immediately.

The analytical typology of van Teijlingen [7], enables us to characterise diverging maternity care systems. The medical model is the dominant paradigm in modern health care and emphasises the body-mind dualism [8] and the risky nature of childbirth. This biomedical focus is doctor-centred and pregnant women are regarded as passive patients, lacking the knowledge or authority to decide on medical treatment. The social model embraces the holistic approach and views birth as a normal physiological process. The medical status of women having children is not the only relevant information, their social roles and status are also taken into account [7]. Manifestations of the social model in the Netherlands are the strong independent midwifery profession [9], the belief in the normality of childbirth [9], the positive attitude towards home births [10], and the low obstetric intervention rates [11,12] compared to other European countries. However, this does not mean that the medical model is completely absent from Dutch maternity care. De Vries [11] points to two sciences of obstetrics in the Netherlands, one in favour of and one against home births. In Belgium the medicalisation of childbirth and the absence of a strong independent midwifery profession [13] translates to the discouragement of home birth practices [5] and high intervention rates. Belgian maternity care, however, does not result in lowered average satisfaction scores in comparison to the Dutch [14].

We assessed four social psychological features – expectations about childbirth, the labour pain, personal control and self-efficacy – associated with childbirth satisfaction in one explanatory model, taking the subdimensions of satisfaction with childbirth into account. Through the use of a Belgian and Dutch sample the applicability of the model in divergent maternity care systems is explored.

### Literature review

Despite a considerable amount of research, satisfaction is poorly defined [15]. Theoretical models regarding patient satisfaction, such as the discrepancy and fulfilment theory [15] and the value-expectancy model [16], are relied on. Following Linder-Pelz [16], we define satisfaction as positive evaluations of distinct dimensions of childbirth. It is generally agreed that satisfaction is a multidimensional concept, influenced by a variety of factors [17]. This means that women can be satisfied with some aspects of childbirth and dissatisfied with others [18]. A review of the literature indicates four main determinants of childbirth satisfaction: labour pain [19-23], personal control [15,20,21,23,24], self-efficacy [25,26] and expectations for labour and birth [19-21].

### Labour pain

Reports about the relationship between the intensity of pain and satisfaction seem to provide mixed results. Some researchers found that painful experiences result in lowered satisfaction [1,19,27-29], others pointed out that the experience of high levels of pain does not necessarily bring about a dissatisfied mother [2,30]. In a longitudinal study assessing the quality of women's birth experience, Doering *et al. *[1] reported that pain does reduce the quality of the birth experience, but even so, remaining in control is more important to a pleasurable experience. In a systematic review Hodnett [18] concluded that pain and pain relief do not play a major role in childbirth satisfaction, unless expectations regarding either are unmet. Apparently, if the question about the influence of the experience of labour pain on satisfaction with childbirth is rigorously reviewed a considerable consensus is reached. Associations between pain intensity and other determinants of satisfaction, e.g., control and the fulfilment of expectations, are suggested.

### Expectations about childbirth

Many authors pointed to the evaluative aspect of childbirth satisfaction [15,16,18,31]. Janzen *et al. *[32] defined satisfaction, corresponding with the "discrepancy" model [17,33], as "the experience which results from the subjective evaluation of the distinction between what actually occurred and what the individual thinks should have" (2006, p. 44).

Expectation as a determinant of satisfaction is related to the need for the familiar, which means that socially created expectations influence satisfaction [34]. Expectations refer to a role system. The role of a labouring woman involves a set of expectations concerning her own behaviour and of people in other roles such as the midwife, the partner, or the physician. By demanding the expected of one's self and each person present, a workable order is created. Violation of expectations disturbs this order and threatens both self-evaluations and relationships with others. In other words, the deviation from what is normal or expected creates distress [35]. Satisfaction is a state of mind reflecting the evaluation of the birth experience as a whole compared with several antenatal values and expectations. If expectations are met, the corresponding values and beliefs are affirmed. If not, conflicts arise, which may bring about distress. However, as Pearlin [36] stated, mediating factors can play a buffering role between the discrepancy and the reaction to it. Personal control is one of those mediators.

Many conceptualisations of satisfaction refer to expectations as a major determining factor of satisfaction [34,37-39]. Researchers have shown that women whose expectations for childbirth are met are more satisfied than those whose expectations are not [2,19,20]. Expectations related to several aspects of labour and delivery, such as emotions [20,40], the length of labour [41], the need for interventions [20,40], the condition of the child [41], and the support of the partner and the medical staff [40], have been researched. Although the fulfilment of expectations received some attention in the childbirth satisfaction literature, it has not yet been included in a model with multiple determinants, except by Goodman *et al.*[2].

### Personal control and self-efficacy

Personal control has been shown to be the strongest predictor of satisfaction with childbirth [2]. Many authors point to the perception of control during birth as essential to feeling satisfied and empowered [1,15,19,20,30,41,42], even if expectations are violated. Although pain management is the best short-term solution to help women cope with childbirth, personal control provides a long-term benefit [30]. If women participate actively, they are empowered by the experience of control [43]. Moreover this empowering experience has a cumulative effect, increasing self-efficacy for the next birth [20]. We distinguished between perceived personal control and self-efficacy. The latter reflects a personality characteristic of confidence in the ability to cope with any stressful situation [44], which predicts a positive childbirth experience [45]. Self-efficacy is also related to lower levels of pain [26,46] and method of delivery [47]. Personal control refers to the opposite of powerlessness, which is a type of alienation [48]. Alienation is thought to be a consequence of the medicalisation of childbirth [49]. The degree of women-centeredness and medicalisation of care varies according to place of birth [50] and the maternity care system [7].

Determinants such as childbirth expectations [51] and personal control [52] have been shown to be strongly related to the birth environment. The results of these studies suggest that the influence of childbirth expectations and personal control can be context specific, hence different for Dutch and Belgian women.

The purpose of our study is to assess the influence of expectations about childbirth, labour pain, personal control and self-efficacy on Belgian and Dutch women's satisfaction with childbirth.

## Methods

### Selection of method

This study modelled the relationships between satisfaction with childbirth and labour pain, expectations about childbirth, personal control and self-efficacy, using data collected from a self-reported survey. To contact as many women as possible in a short period of time, a survey by two questionnaires – one at 30 weeks of pregnancy and one within 2 weeks postpartum – was considered to be appropriate. Because of the longitudinal design the same concepts were measured before and after birth, hence the antenatal and postnatal questionnaires were similar. From the time the invitation to participate was issued, to the completion of the last questionnaire, five to eight months passed. Since the data collection was not simultaneously organised in each hospital/midwifery practice, one year – from September 2004 to September 2005 – was necessary to gather the data.

### Settings

Satisfaction with childbirth was studied in two different health care contexts, namely Belgium and the Netherlands. The Netherlands are well-known as an important exception to the worldwide trend of institutionalisation and medicalisation of childbirth [53]. No other region resembles the Dutch society more closely than Belgium does. Still, Belgian maternity care is strikingly different. The study concerned two comparable cities in the Belgian and Dutch regions, respectively. To enhance the readability of the paper we will refer to Belgium and the Netherlands, and the Belgians and the Dutch. Both hospital and home births are included, because it has been shown that place of birth influences satisfaction with childbirth [52].

### Sample size

Since the population of pregnant women is unknown, we had to rely on a convenience sample. With regard to the hospital births all hospitals in both cities were approached. In Ghent there are four hospitals of which three agreed to participate. We have no reasons to believe that the population of the missing hospital differs from the population in the participating hospitals. In Tilburg both hospitals agreed to cooperate. Since there are more hospital than home births in both countries, we needed to over sample the home deliveries. In Tilburg six midwifery practices were contacted to reach enough women planning a home delivery. Ghent does not count enough midwifery practices to attain the same number of home births. Therefore, the city borders of Ghent were exceeded and 21 midwifery practices spread out over Flanders were contacted. This was necessary to compare the four kinds of birth settings determined by country (Belgium versus the Netherlands) and place of birth (home versus hospital births). Sample size calculations based on a 0.95 confidence interval suggested that 600 study participants were needed for a reliable statistical analysis. At 30 weeks of pregnancy, 827 women filled out the antenatal questionnaire; 605 of those women also participated in the study in the first 2 weeks after delivery and completed a second questionnaire.

### Recruitment and data collection

During antenatal visits, women were asked by their midwife or obstetrician to participate in the research project. Inclusion criteria were wide: both Belgian and Dutch women had to speak and understand Dutch, and had to be 18 years or older. The antenatal questionnaire was handed out during an antenatal visit at 30 weeks of pregnancy together with a prepaid envelope and an information sheet. It was returned to the obstetrician or midwife during one of the following antenatal visits. Within a few days after delivery, women received the postnatal questionnaire from the medical staff in case of a hospital birth, or from the midwife in case of a home birth. Women who delivered in a hospital completed the postnatal questionnaire during their postpartum stay on the maternity ward. Women with a short stay or home birth, however, responded by direct mail instead. Ante- and postnatal questionnaires were given a code, to facilitate the merging of the ante- and postnatal information belonging to the same respondent.

Women were recruited during antenatal visits to their obstetricians and midwives. Therefore, we had little control over the inclusion process and, consequentially, the response rate. Although we asked that women who refused to participate be registered, this was not systematically done in every hospital. As a result, we do not know the exact number of women invited to participate in this study. To calculate the response rate we used the number of provided questionnaires, which is based on an estimate of eligible women made by midwives and obstetricians acting as proxy. The response rate is calculated by dividing the number of respondents by the number of provided questionnaires. This calculation resulted in an average of 43% (n = 238) for all Belgian hospitals, 41% (n = 137) for Belgian midwifery practices, 42% (n = 208) for Dutch hospitals, and 54% (n = 244) for Dutch midwifery practices. The smallest response rate for the hospitals was 19%, the highest 68%. For the midwifery practices the response rate was 38% and 100% respectively.

### Ethical considerations

A written informed consent was asked of all respondents. Anonymity has been guaranteed, since the researchers have no information about the identity of the respondent. The Committee for Ethics of the University Hospital has approved the study. Ethical approval was gained in Ghent only. In the Netherlands, approval from a research Ethics committee is not required if no interventions take place during the research. It has been explained to potential participants that they were free to participate and that their privacy was guaranteed.

### Measurement

#### Dependent variables

Satisfaction is measured by the Mackey Childbirth Satisfaction Rating Scale, which consists of six subdimensions – general satisfaction (three items) and satisfaction with self (nine items), baby (three items), midwife (nine items), physician (eight items), and partner (two items) – thus reflecting the multidimensional nature of the concept. Each dimension corresponds to a separate dependent variable in our analysis. The scale was designed by M. Mackey and P. Goodman [2]. The scale was translated for Belgian and Dutch women. Pilot testing demonstrated that the instrument was valid. The sample Goodman *et al. *[2] used was limited to low-risk postpartum women with uneventful vaginal deliveries, whereas our sample extends the scope to women with instrument deliveries. Respondents indicate their degree of satisfaction with each item on a 5-point Likert scale. Internal consistency reliability coefficients (Cronbach's alpha for N = 605) for this study (total scale: *α *= 0.95; self: *α *= 0.84; baby: *α *= 0.74; midwife: *α *= 0.96; physician: *α *= 0.94; partner: *α *= 0.85; general: *α *= 0.71) are similar to those established by Goodman *et al. *[2] (total scale: *α *= 0.94; self: *α *= 0.90; baby: *α *= 0.70; midwife: *α *= 0.97; physician: *α *= 0.83; partner: *α *= 0.97; and, general: *α *= 0.93). This means that the items measuring one subscale cluster together in the translated version to the same extent as in the original English version of the scale. For each subscale, means were calculated.

#### Independent variables

Two Visual Analogue Scales (VAS) – one about labour and one about delivery – measured the experience of pain, ranging from no pain at all (0) to unbearable pain (100). Mean scores were calculated to merge both scales to one indicator of pain intensity. The measurement of labour pain by visual analogue scales is common practice in research on childbirth [54-56] and has been found to be reliable for estimating pain intensity. In comparison to more complex pain measures, the VAS is preferable [57,58].

To measure personal control, three items from the Wijma Delivery Expectancy/Experience Questionnaire (W-DEQ) were isolated (Cronbach's *α *= 0.67). The items are "I behaved extremely badly" to "I didn't behave badly at all"; "I dared to totally surrender control to my body" to "I did not dare surrender control to my body at all"; "I lost total control of myself" to "I did not lose control of myself at all", and they were scored from zero to six [59]. The W-DEQ was developed in Dutch to measure fear related to childbirth by assessing women's expectations before and experiences after childbirth. Because the entire scale is too broad in scope and shows overlap with the pain measure, only the control-related items were isolated in order to assess the control experience during delivery. In addition we used the seven-item mastery scale developed by Pearlin and Schooler [60] to measure self-efficacy. The seven items are: "I have little control over the things that happen to me", "There is really no way I can solve some of the problems I have", "There is little I can do to change many of the important problems I have", "I often feel helpless in dealing with the problems of life", "Sometimes I feel that I'm being pushed around in life", "What happens to me in the future mostly depends on me", and "I can do just about anything I really set my mind to do"; each item has five answer options ranging from 'strongly agree' to 'strongly disagree'. The psychometric properties of the Dutch version of this scale have been successfully tested in a study by Kempen [61]. The reliability of this scale as measured by Cronbach's alpha was 0.79.

The degree to which expectations concerning childbirth are fulfilled was measured by the question, "To what degree was your experience of childbirth as expected?" The four answer options ranged from "not at all" to "completely in accordance with my expectations".

We controlled for childbirth characteristics such as length of labour, the planned place of birth and the method of delivery. The length of labour was a self-reported indicator. Respondents filled in when labour started and when the baby was born. The method of delivery gave an indication of how the child was born: spontaneously (= 0) or with a medical intervention such as a C-section, a vacuum extraction or a forceps delivery (= 1). We asked for the intended place of birth in the antenatal questionnaire using the following question: *Where would you like to give birth? *Answer categories were: *in hospital, policlinical, at home, in a birth clinic, other, I don't know*. Hence this variable consisted of two broad categories, the *home *(= 0) versus the *hospital *(= 1), as intended place of birth. Women planning for a birth in a birth clinic were considered primary care clients, because a birth clinic is a substitution for the home and is not considered a medically sophisticated environment. Planning for a policlinical birth or short stay was coded as a hospital birth, notwithstanding that in some cases only midwives provided care. Nobody had a place in mind other than the ones summed up. Women who had not yet made up their minds about the place of birth (N = 6) were coded as missing value. Following De Vries and Lemmens [62] we based the analysis on planned rather than actual place of birth, because the most complicated births end up in a hospital. This strategy avoids a positive bias towards home births and a negative bias towards hospital births.

Also socio-demographic characteristics were included as control variables: level of education (0 = no higher education; 1 = higher education), marital status (0 = married/cohabiting; 1 = single), parity (0 = primiparous; 1 = multiparous), age in years, and employment status (0 = unemployed; 1 = employed).

### Data analysis

To explore the data, descriptive statistics and correlations among the study variables were reported. Because the dataset is hierarchically structured, and in order to control for clustering of women within countries, a hierarchical linear model with women (first level) nested within countries (second level) was fitted to the data. Multilevel models take into account dependence among cases from the same context to produce parameter estimates and standard errors that are more accurate. Estimations were performed using the mixed model procedure of SPSS 12.0 for total childbirth satisfaction and each subdimension, using restricted maximum likelihood estimation [63]. Two models were estimated. The first model contains the main effects of the social psychological determinants – labour pain, personal control, self-efficacy and the fulfilment of expectations – together with childbirth characteristics – place of birth, method of delivery and length of labour – to estimate the main effects. The regression equation of this model on the level of individual women is

*Y*_*ij *_= *β*_0*j *_+ *β*_1*j*_(*Education*_*ij*_) + *β*_2*j*_(*Maritalstatus*_*ij*_) + *β*_2*j*_(*Parity*_*ij*_) + *β*_3*j *_(*Age*_*ij*_) + *β*_4*j *_(*Employment*_*ij*_) + *β*_5*j*_(*Length of lobour*_*ij*_) + *β*_6*j *_(*Placeof birth*_*ij*_) + *β*_7*j*_(*Methodof delivery*_*ij*_) + *β*_8*j*_(*Labour pain*_*ij*_) + *β*_9*j *_(*Personalcontrol*_*ij*_) + *β*_10*j *_(*Self *- *efficacy*_*ij*_) + *β*_11*j *_(*Expectations*_*ij*_) + *β*_12*j *_(*Personalcontrol*_ij _* *Labour Pain*_*ij*_) + *r*_*ij*_

where *Y*_*ij *_is one of the subdimensions of satisfaction with childbirth (total, general, self, baby, midwife, physician, partner) of women i in county j experienced shortly after birth, *β*_0*j *_is the women-level intercept; *β*_1*j *_to *β*_12*j *_are the effects of the control variables, characteristics of childbirth and social-psychological determinants of satisfaction, and *r*_*ij *_is the error term.

At the country level the model is

*β*_0*j *_= *γ*_00 _+ *u*_0*j*_

where *γ*_00 _is the organization-level intercept and *u*_0*j *_is the error term. No effects of country-level characteristics are included. Substituting the country-level equation into the individual-level equation gives the combined model in the following equation

*Y*_*ij *_= *γ*_00 _+ *β*_1*j*_(*Education*_*ij*_) + *β*_2*j*_(*Maritalstatus*_*ij*_) + *β*_2*j*_(*Parity*_*ij*_) + *β*_3*j *_(*Age*_*ij*_) + *β*_4*j *_(*Employment*_*ij*_) + *β*_5*j*_(*Length of lobour*_*ij*_) + *β*_6*j *_(*Placeof birth*_*ij*_) + *β*_7*j*_(*Methodof delivery*_*ij*_) + *β*_8*j*_(*Labour pain*_*ij*_) + *β*_9*j *_(*Personalcontrol*_*ij*_) + *β*_10*j *_(*Self *- *efficacy*_*ij*_) + *β*_11*j *_(*Expectations*_*ij*_) + *β*_12*j *_(*Personalcontrol*_ij _* *Labour Pain*_*ij*_) + *u*_0*j *_+ *r*_*ij*_

To test whether the determinants apply equally to both Belgian and Dutch women, a second model containing between-county interaction terms of the social psychological determinants and the childbirth characteristics was included. In both models, level of education, marital status, parity, age and employment status were controlled for in this analysis. No random effects were included in the model structure. Because we fitted a parsimonious model, non-significant interactions with country were not included in the final model.

## Results

Within the first 2 weeks after delivery, 605 women, of which 261 are Belgian and 344 are Dutch, filled out a questionnaire. In our analysis we focused on this follow-up data. The number of cases in the analysis dropped to 560 because 51 women failed to provide information on one of the determinants in the model. For the subscale of satisfaction with the doctor the number of cases dropped to 393 because women with a home birth did not see a physician and therefore did not answer the physician-related items.

### Descriptives

The age of participating women ranged between 19 and 44 years, with a mean of 31.2 years, 30.4 for Belgian women and 31.9 for Dutch women. Those having their first baby made up 54.2% of all respondents, with 42.2% in Belgium and 51.8% in the Netherlands. Approximately 98.0% of the respondents were married or living as married in both Belgium and the Netherlands. More Belgian (76.9%) than Dutch (40.5%) women have completed higher education, and 85.3% of all women were employed, with 85.3% in Belgium and 84.8% in the Netherlands (Table 1). This means parity and educational level may confound the comparison between Belgium and the Netherlands. Therefore these variables were controlled for in the hierarchical linear model.

**Table 1:** Descriptive statistics in total and for Belgian and Dutch women separately

		***Total***	***Belgium***	***The Netherlands***
***Socio-demographic variables***				

Higher education	%	57.11	76.99	40.50
	n	329	194	135
	CI	0.55 – 0.59	0.75 – 0.79	0.38 – 0.43
Married/cohabitating	%	98.40	97.90	98.90
	n	596	257	339
	CI	0.976 – 0.988	0.978 – 0.979	0.988 – 0.989
Primiparae	%	54.20	48.20	51.80
	n	276	133	143
	CI	0.51 – 0.55	0.45 – 0.51	0.48 – 0.54
Employed	%	85.28	85.23	84.82
	n	517	221	296
	CI	0.84 – 0.87	0.84 – 0.88	0.83 – 0.87
Age	Mean	31.21	30.41	31.87
	SD	4.17	4.09	4.14
	n	816	372	444
	CI	31.06 – 31.36	30.20 – 30.62	31.68 – 32.07

***Characteristics of childbirth***				

Length of labour (expressed in hours)	Mean	9.48	9.95	9.13
	SD	6.31	6.13	6.44
	CI	9.22 – 9.74	9.57 – 10.34	8.79 – 9.47
With medical intervention (1)	%	22.50	20.80	23.90
	n	133	54	79
	CI	0.21 – 0.24	0.18 – 0.23	0.22 – 0.26
Planning for a home birth (0)	%	37.00	24.00	48.00
	n	301	90	211
	CI	0.35 – 0.39	0.22 – 0.26	0.46 – 0.50

***Social psychological variables***				

Labour pain	Mean	57.55	58.04	57.55
	SD	22.02	22.33	21.67
	CI	56.66 – 58.45	56.67 – 59.42	56.38 – 58.72
Personal control	Mean	4.34	4.27	4.37
	SD	1.24	1.19	1.29
	CI	4.28 – 4.39	4.20 – 4.35	4.30 – 4.44
Self-efficacy	Mean	3.89	3.89	3.89
	SD	0.53	0.53	0.53
	CI	3.87 – 3.91	3.86 – 3.92	3.87 – 3.92
Expectations met	Mean	2.59	2.69	2.50
	SD	0.96	0.93	0.97
	CI	2.55 – 2.63	2.63 – 2.75	2.44 – 2.55

The mean length of labour in our sample was approximately 10 hours for both Belgian and Dutch women. Of the respondents, 22.5% had a medical intervention. The mean pain experience was 57.6 (max. = 100). The moderate score is a result of the pain medications used by 32.8% of the respondents. The mean pain score for women who gave birth without painkillers is 62.1. A total of 64.8% reported that their expectations about childbirth were met (Table 1).

Overall women reported a high childbirth satisfaction (mean = 4.21; SD = 0.53; max. = 5), but the mean scores differed along the subscales (Table 2). Satisfaction with self-related aspects of childbirth was lowest (mean = 3.81; SD = 0.71), while satisfaction with partner-related aspects was highest (mean = 4.65; SD = 0.53).

**Table 2:** Means, skewness and kurtosis for each subdimension of the Mackey Childbirth Satisfaction Rating Scale in total and for Belgian and Dutch women (N = 605)

***Satisfaction with childbirth***		***Total***	***Belgium***	***The Netherlands***	***Potential min. – max Real min. – max.***
Total	Mean (SD)	4.21 (0.53)	4.37 (0.46)	4.08 (0.54)	1.00 – 5.00
	Skewness (SE)	-0.97 (0.10)	-1.34 (0.15)	-0.77 (0.13)	2.15 – 5.00
	Kurtosis (SE)	1.04 (0.20)	3.25 (0.30)	0.37 (0.26)	
General	Mean (SD)	4.03 (0.72)	4.14 (0.68)	3.93 (0.74)	1.00 – 5.00
	Skewness (SE)	-0.86 (0.10)	-1.00 (0.15)	-0.75 (0.13)	1.00 – 5.00
	Kurtosis (SE)	0.80 (0.20)	1.13 (0.30)	0.63 (0.26)	
Self	Mean (SD)	3.81 (0.71)	3.99 (0.66)	3.67 (0.72)	1.00 – 5.00
	Skewness (SE)	-0.65 (0.10)	-0.81 (0.15)	-0.58 (0.13)	1.25 – 5.00
	Kurtosis (SE)	0.34 (0.20)	1.24 (0.30)	-0.04 (0.26)	
Baby	Mean (SD)	4.39 (0.77)	4.48 (0.77)	4.34 (0.76)	1.00 – 5.00
	Skewness (SE)	-1.63 (0.10)	-2.00 (0.15)	-1.42 (0.13)	1.00 – 5.00
	Kurtosis (SE)	2.98 (0.20)	4.52 (0.30)	2.34 (0.26)	
Midwife	Mean (SD)	4.46 (0.66)	4.62 (0.55)	3.32 (0.73)	1.00 – 5.00
	Skewness (SE)	-1.89 (0.10)	-2.91 (0.15)	-1.44 (0.13)	1.00 – 5.00
	Kurtosis (SE)	4.68 (0.20)	13.04 (0.30)	2.40 (0.26)	
Physician	Mean (SD)	4.20 (0.75)	4.36 (0.72)	4.07 (0.75)	1.00 – 5.00
	Skewness (SE)	-1.29 (0.12)	-1.98 (0.76)	-0.88 (0.14)	1.00 – 5.00
	Kurtosis (SE)	2.14 (0.24)	5.38 (0.35)	0.85 (0.33)	
Partner	Mean (SD)	4.65 (0.53)	4.74 (0.46)	4.59 (0.57)	1.00 – 5.00
	Skewness (SE)	-1.52 (0.10)	-1.53 (0.15)	-1.419 (0.134)	1.50 – 5.00
	Kurtosis (SE)	2.44 (0.20)	1.19 (0.30)	2.424 (0.268)	

### Hierarchical linear model

The results of seven hierarchical analyses are summarised in Table 3. We will first concentrate on the main effects, and continue further on with the country-specific effects (no table). We present the regression coefficients (B) and the significance of the findings (P-value) between brackets. Standardised regression coefficients (*β*) can be found in table 3. First, total satisfaction benefited from a feeling of being in control (B = 0.14, *P *< 0.001) and from a high degree of self-efficacy (B = 0.14, *P *< 0.001). Also the fulfilment of expectations (B = 0.13, *P *< 0.001) improved total childbirth satisfaction, whereas this was not the case for perceived pain (B = -0.001, *P *= 0.341). Second, we consider the subdimensions of satisfaction with childbirth. General satisfaction was improved by every social psychological determinant (expectations: B = 0.24, *P *< 0.001; personal control: B = 0.15; *P *< 0.001; self-efficacy: B = 0.11; *P *= 0.017) in our model, except for labour pain (B = -0.01, *P *< 0.001), which had a small lowering effect. Issues of control and decision-making were central to the self-related satisfaction scores. Not surprisingly, self-related satisfaction was enhanced by feelings of control (B = 0.23, *P *< 0.001) and self-efficacy (B = 0.18, *P *< 0.001). Also, one's own performance was more positively evaluated when expectations were met (B = 0.17, *P *< 0.001). Equally, the first contact with the baby was more satisfying if it corresponded with expectations (B = 0.15, *P *< 0.001). The same (B = 0.10, *P *= 0.001) applies for the midwife, who was more positively evaluated by women with high self-efficacy (B = 0.10, *P *= 0.041). Personal control interacted with labour pain (B_pain*control _= 0.002, *P *= 0.012), which means that women were generally satisfied about the midwife when they felt in control, even when suffering from serious labour pain, but the B-coefficient is very small. The evaluation of the physician was positively influenced by the self-efficacy (B = 0.19, *P *= 0.008) of the women, whereas pain intensity (B = 0.003, *P *= 0.107) and the fulfilment of expectations (B = 0.08, *P *= 0.069) were of no importance. The more pain (B = 0.002; *P *= 0.036) and personal control (B = 0.052; *P *= 0.008) women experienced during childbirth, the more satisfied they were with the support of their partner, but again these relations were weak.

**Table 3:** Coefficients for a hierarchical linear model of the determinants of satisfaction with childbirth

		Total (N = 546)			General (N = 546)			Self (N = 545)			Baby (N = 546)			Midwife (N = 541)			Physician (N = 379)			Partner (N = 530)		
			CI (95%)			CI (95%)			CI (95%)			CI (95%)			CI (95%)			CI (95%)			CI (95%)	

Intercept	B	2.885	2.367	3.402	2.676	1.977	3.375	1.881	1.210	2.551	3.559	2.717	2.551	4.334	3.374	5.294	2.333	1.355	3.311	4.272	3.612	4.932
	SE	0.263			0.356			0.341			0.428			0.489			0.497			0.336		
Length of labour	B	-0.006	-0.012	0.000	-0.016**	-0.024	-0.0084	-0.004	-0.011	0.004	-0.007	-0.016	0.003	-0.004	-0.012	0.005	-0.010	-0.022	0.001	0.001	-0.006	-0.009
	*β*	-0.068			-0.140			-0.031			-0.055			-0.036			-0.087			0.015		
	SE	0.003			0.004			0.004			0.005			0.004			0.006			0.004		
Place of birth	B	-0.166***	-0.238	-0.093	-0.061	-0.160	0.037	-0.259***	-0.354	-0.165	-0.100	-0.218	-0.165	-0.190**	-0.298	-0.082	0.113	-0.058	0.285	-0.077	-0.170	0.017
	*β*	-0.13			-0.035			-0.152			-0.054			-0.120			0.063			-0.061		
	SE	0.037			0.050			0.048			0.060			0.055			0.087			0.047		
Method of delivery	B	-0.185***	-0.277	-0.094	-0.220**	-0.344	-0.096	-0.123*	-0.242	-0.004	-0.660***	-0.809	-0.004	-0.176*	-0.313	-0.040	-0.072	-0.238	0.093	-0.041	-0.164	0.082
	*β*	-0.169			-0.148			-0.084			-0.414			-0.129			-0.046			-0.037		
	SE	0.047			0.063			0.061			0.076			0.069			0.084			0.063		
Labour pain	B	-0.001	-0.003	0.001	-0.005***	-0.007	-0.003	-0.003*	-0.005	-0.001	-0.001	-0.004	-0.001	-0.011*	-0.020	-0.002	0.003	-0.001	0.006	0.002*	0.000	0.005
	*β*	-0.042			-0.153			-0.093			-0.029			-0.367			0.088			0.083		
	SE	0.001			0.001			0.001			0.001			0.005			0.002			0.001		
Personal control	B	0.142***	0.113	0.172	0.148***	0.108	0.188	0.230***	0.192	0.268	0.004	-0.044	0.268	-0.024	-0.150	0.102	0.118***	0.058	0.179	0.052**	0.014	0.090
	*β*	0.332			0.255			0.402			0.006			-0.045			0.195			0.122		
	SE	0.015			0.020			0.020			0.025			0.064			0.031			0.019		
Self-efficacy	B	0.135***	0.068	0.203	0.111*	0.020	0.203	0.181***	0.093	0.269	0.082	-0.029	0.269	0.104*	0.004	0.205	0.191**	0.051	0.332	0.081	-0.006	0.169
	*β*	0.135			0.082			0.135			0.056			0.084			0.135			0.081		
	SE	0.035			0.047			0.045			0.056			0.051			0.071			0.045		
Expectations met	B	0.126***	0.086	0.165	0.243***	0.189	0.296	0.169***	0.118	0.220	0.145***	0.080	0.220	0.101**	0.042	0.159	0.075	-0.006	0.155	-0.002	-0.054	0.049
	*β*	0.228			0.324			0.229			0.181			0.147			0.096			-0.004		
	SE	0.020			0.027			0.026			0.033			0.030			0.041			0.026		
Country	B	-0.348***	-0.428	-0.269	-0.194***	-0.301	-0.086	-0.404***	-0.507	-0.301	-0.190**	-0.319	-0.301	-0.362***	-0.480	-0.245	-0.296***	-0.462	-0.131	-0.174**	-0.276	-0.071
	*β*	-0.327			-0.134			-0.283			-0.123			-0.273			-0.197			-0.163		
	SE	0.040			0.055			0.052			0.066			0.060			0.084			0.052**		
Personal control*labour pain	B													2.551	0.001	0.004						
	SE	.	.	.	.	.	.	.	.	.	.	.	.	0.001			.	.	.	.	.	.

R^2^		0.36			0.40			0.42			0.24			0.13			0.08			0.03		
-2 restricted loglikelihood		664.58			993.26			946.62			1196.1			1196.1			1092.12			894.8		

* p < 0.05; ** p < 0.01; *** p < 0.001

In addition we want to mention that women having birth in hospital reported lower satisfaction in total (B = -0.17, *P *< 0.001), and for some of the subdimensions (self: B = -0.26, *P *< 0.001; midwife: B = -0.19, *P *= 0.001). Longer labours resulted in lower general satisfaction, although the association is weak (B = -0.016, *P *< 0.001). Total satisfaction and most subdimensions of satisfaction decreased in cases where medical interventions occurred (Total: B = -0.19; *P *< 0.001; general: B = -0.22; *P *= 0.001; self: B = -0.123; *P *= 0.044; baby: B = -0.66; *P *< 0.001; midwife: B = -0.18; *P *= 0.011). Finally, parity (no table) was associated with general satisfaction (B = 0.14, *P *= 0.014) and with self (B = 0.12, *P *= 0.019) and baby-related (B = 0.16, *P *= 0.015) satisfaction, indicating that multiparous mothers tended to be more satisfied.

Additional analyses (no table) learned that, in general, the abovementioned results apply equally to Belgian and Dutch women. Nevertheless, two country-specific effects occurred. First, results showed that women with home births were more satisfied, especially in Belgium (general: B_place*country _= 0.35, *P *< 0.001; self: B_place*country _= 0.43, *P *< 0.001). This 'place of birth'*'country' interaction effect totally explained the between-country differences in self-related satisfaction. Second, Dutch women's self-related satisfaction (B_control*country _= 0.08, *P *= 0.040) was lower than that of Belgian women, especially when they experienced control loss, but the coefficient was small. Despite significant p-values, these country-specific effects added little to the model, since the likelihood ratio test (p_general _> 0.25; p_self _> 0.10) was not significant.

The variance of each outcome of the Mackey Childbirth Satisfaction Rating Scale explained by covariates, ranges between 3% of satisfaction with the partner's support and 42% for self-related aspects of the birth experience (Table 3).

In sum, the fulfilment of expectations is the most consistent determining factor across the subdimensions of satisfaction with childbirth, except for physician- and partner-related satisfaction. The more expectations were met, the more women were satisfied. Another important factor is the experience of personal control, which buffered even the lowering impact of labour pain for midwife-related satisfaction. Finally, women with high self-efficacy showed more satisfaction with their own performance, as well as the support of midwife and physician. The model is applicable to the satisfaction scores of both Belgian and Dutch women.

## Discussion

In a sample of 311 Dutch and 249 Belgian women, we tested a model including four social psychological determinants of satisfaction with childbirth which have been the subject of other childbirth satisfaction research: the experience of labour pain [19-23], personal control [15,20,21,23,24], self-efficacy [25,26] and the fulfilment of expectations [19-21]. Characteristics of childbirth (such as intended place of birth, length of labour, method of delivery) and of the mother (such as age, parity, level of education, marital status and employment) were controlled for.

Before further discussing the findings, we want to briefly list some of the shortcomings and merits of the study. Weaknesses of our research relate to the timing of the measurement of satisfaction with childbirth. Questionnaires were answered within 2 weeks after delivery. This close to the birthing experience, women might have answered less critically than they would have later on [64]. However, the two-week time frame applied to all respondents and therefore does not affect the differences between the groups compared. Second, comparability of the Dutch and Belgian sample can be questioned: Belgian women were on the average more highly educated, younger at first birth and more likely to give birth for the first time in comparison to the Dutch. The higher education of the Belgian sample can be explained by the over sampling of home births, since in Belgium women preferring a home birth are on the average more highly educated [5]. In the Netherlands women are on the average older at first birth in comparison to Belgium and the rest of Europe [65]. Age and education are controlled for in the analysis. Third, women who refused to participate were not systematically registered. This makes generalization of the results less likely.

Despite the limitations, the inclusion of multiple determinants – labour pain, personal control, self-efficacy and the fulfilment of expectations – into one model proved to be fruitful in explaining satisfaction with childbirth. In addition, we used the Mackey Childbirth Satisfaction Rating Scale to take the multidimensionality of childbirth satisfaction into account. Finally, by estimating the models in both the Belgian and the Dutch sample, we tried to assess the applicability of the model for Belgian and Dutch women. Because both countries have strongly differing maternity care systems, we are confident that the present findings have a more general meaning.

Four important findings arise from this investigation. First, the fulfilment of expectations was the most consistent determining factor of satisfaction with childbirth. Women whose expectations for childbirth were met were more satisfied than those whose expectations were not. This conclusion corresponds to the conceptualisation of satisfaction and confirms previous research [2,19,20]. Moreover, by comparing Belgian and Dutch women, we learned that the fulfilment of expectations is equally important to the childbirth satisfaction of both groups. However, this does not exclude the possibility that Dutch and Belgian women's expectations may differ. This is rather likely because of the diverging maternity care systems. Expectations are context specific, but the association between their fulfilment and satisfaction is not. In addition, we learned from this study that Belgian women's expectations were more easily fulfilled than Dutch women's expectations. This is not surprising when taking the high referral rate into account. Nearly one third of all planned home deliveries end up in hospital [66]. The ambivalent Dutch maternity care, with its two sciences of obstetrics [11], might explain the unfulfilled Dutch expectations. In ambivalent social structures, contrary courses of action are simultaneously valued for a single actor in a given situation [67]. The conflicting normative expectations imposed on Dutch women may result in a decreased childbirth satisfaction, because it is impossible to conform, without being deviant at the same time.

Second, we found that personal control consistently improved satisfaction and buffered the lowering impact of labour pain. The latter mediating effect is limited to satisfaction with the midwife's support, but nevertheless it supports conclusions of Doering *et al. *[1] and Pellino and Ward [68] and points to the importance of including personal control and labour pain in one analysis. This interplay between labour pain and control might explain the lack of consensus about the relationship between labour pain and childbirth satisfaction.

Third, women with high self-efficacy showed more satisfaction, especially with the support of the midwife and the physician. This result corresponds with the findings of Crowe and von Baeyer [45] about self-efficacy leading to positive birth experiences.

Personal control and self-efficacy are mediators in the stress process, as predicted by the social stress model [36]. A demanding birth does not result in dissatisfaction if women keep control, hence feel empowered. The job strain model [69] designed to explain the impact of work-related stress on health outcomes can equally be applied to childbirth satisfaction. The model postulates that strain results from the joint effects of the demands of a work situation, and the control workers facing those demands, exert [69]. The practical implication of the model is that in redesigning maternity care an increase in personal control can be pursued, even without affecting the demanding nature of birth itself. In other words, the empowerment of labouring women, not the management of childbirth by means of painkillers, leads to satisfactory birth experiences.

Finally, the model explains the satisfaction scores of Belgian and Dutch women, implying that the social psychological determinants affect satisfaction independently of the context in which they operate.

## Conclusion

Our findings focus attention toward personal control, self-efficacy and expectations about childbirth. Antenatal preparation could enhance mother's satisfaction with childbirth by providing techniques for maintaining control, by the enhancement of self-efficacy and by providing information that gives way to realistic expectations about childbirth. This study confirms the multidimensionality of childbirth satisfaction and demonstrates that different factors predict the various dimensions of satisfaction. Further research should incorporate other potential predictors of satisfaction with childbirth, such as social support. Additionally, cross-national comparative research should further assess the dependence of the determinants of childbirth satisfaction on national organisation of maternity care.

## Competing interests

The author(s) declare that they have no competing interests.

## Authors' contributions

WC carried out the literature review, collected the data, interpreted the results and drafted the manuscript; PB participated in the design of the study, performed statistical analyses and revised the manuscript. Both authors read and approved the final manuscript.

## Pre-publication history

The pre-publication history for this paper can be accessed here:


